# TIMSImaging: an open and interoperable workflow for trapped ion mobility mass spectrometry imaging data processing and visualization

**DOI:** 10.1093/bioinformatics/btag624

**Published:** 2026-08-25

**Authors:** Yinyue Zhu, Kylie Ariel Bemis, Sai Srikanth Lakkimsetty, Mujia Jenny Li, Larissa Chiara Meyer, Andreas Weber-Steinhilber, Melanie Christine Föll, Olga Vitek

**Affiliations:** Khoury College of Computer Sciences, Northeastern University, Boston, MA, United States; Barnett Institute for Chemical and Biological Analysis, Northeastern University, Boston, MA, United States; Khoury College of Computer Sciences, Northeastern University, Boston, MA, United States; Barnett Institute for Chemical and Biological Analysis, Northeastern University, Boston, MA, United States; Khoury College of Computer Sciences, Northeastern University, Boston, MA, United States; Barnett Institute for Chemical and Biological Analysis, Northeastern University, Boston, MA, United States; Institute of Surgical Pathology, University of Freiburg, Faculty of Medicine, Freiburg, Germany; Institute for Pharmaceutical Sciences, University of Freiburg, Freiburg, Germany; Institute of Surgical Pathology, University of Freiburg, Faculty of Medicine, Freiburg, Germany; Faculty of Biology, University of Freiburg, Freiburg, Germany; Institute of Surgical Pathology, University of Freiburg, Faculty of Medicine, Freiburg, Germany; Institute of Surgical Pathology, University of Freiburg, Faculty of Medicine, Freiburg, Germany; Khoury College of Computer Sciences, Northeastern University, Boston, MA, United States; Barnett Institute for Chemical and Biological Analysis, Northeastern University, Boston, MA, United States

## Abstract

**Summary:**

Mass spectrometry imaging (MSI) enables high-throughput spatial mapping of molecules, but the lack of chromatographic separation limits its utility for complex biological samples. Ion mobility (IM) provides key orthogonal separation. However, open-source tools dedicated for IM-MSI data analysis remain scarce and the integration of ion mobility information into downstream analysis is underexplored. Here, we present TIMSImaging, an open-source workflow for processing and visualization of MALDI-TIMS-MS data from Bruker timsTOF instruments. TIMSImaging incorporates a graph-based two-dimensional feature extraction algorithm for separation of isobaric peaks by ion mobility, supports collision cross section (CCS) calculation, and exports results as imzML files with ion mobility for downstream analysis. We demonstrate its capabilities on three case studies spanning different sample types, analyte types, and downstream tasks.

**Availability and implementation:**

TIMSImaging is released as open-source software under the MIT License. The source code, installation instructions, documentation, and case study Vignettes are available at https://github.com/YinyueZhu/TIMSImaging

## 1 Introduction

Mass spectrometry imaging (MSI) enables high-throughput mapping of molecular distributions in biological tissues, and has become a central technique in spatial metabolomics, lipidomics, and proteomics (Djambazova *et al*. 2020, Ma and Fernandez 2024, Zhang *et al*. 2024). However, a major limitation of MSI is the lack of an orthogonal separation dimension, such as liquid chromatography. As a result, isobaric and isomeric species cannot be distinguished and most acquisitions remain at the MS1 level, limiting confident identification and molecular interpretation (Alexandrov 2020).

Ion mobility (IM) provides an additional gas-phase separation dimension that is compatible with MSI. In an electric field, ions are separated based on their mobility in a buffer gas, which depends on their mass, charge, structure, as well as buffer gas and other experimental conditions. The measurement is usually recorded as inverse reduced values ($1/K_{0}$), which is independent of experiment pressure and temperature. When coupled with MSI, IM-MSI increases peak capacity, dynamic range, and molecular specificity in complex samples (Paglia *et al*. 2022, Liu *et al*. 2025), and further enables targeted on-tissue MS2 level imaging using iprm-PASEF (Li *et al*. 2025, Shepherd *et al*. 2025). In addition, collision cross section (CCS) values can be derived from ion mobility, providing molecular descriptors that facilitate cross-platform annotation and structural interpretation (Picache *et al*. 2019, George *et al*. 2024).

IM-MSI technologies have advanced substantially over the past decade, with several commercial platforms now available, including MALDI-TIMS-MSI (Spraggins *et al*. 2019) and DESI-cIMS-MSI (Giles *et al*. 2019). However, the resulting multi-dimensional data introduces significant computational challenges for analysis. Current workflows (Leontyev *et al*. 2024, Ngai *et al*. 2024) rely heavily on vendor-provided software with limited accessibility, be it cost, closed-source or propreitary formats, and the lack of open-source tools with ion mobility support has become a major bottleneck for flexible and reproducible analysis.

To address this challenge, we developed TIMSImaging, an open workflow for processing and visualization of MALDI-TIMS-MSI data. TIMSImaging enables IM-aware spectral processing, interactive visualization, CCS calculation and export of imzML with ion mobility dimension. In addition, we present three case studies that illustrate the compatibility of IM-resolved data in downstream analyses, and the benefits of the additional ion mobility information.

## 2 Background

A typical MSI dataset can be represented as a set of mass spectra, each acquired at an *x*-*y* position. After spectral processing, the data can be interpreted as a hyperspectral image datacube, where each channel maps ion intensities at a specific mass-to-charge (m/z) value.

Supplemental Table S1, available as supplementary data at *Bioinformatics* online summarizes the tools related to MSI data analysis. Traditionally, data analysis relies on vendor-provided software tied to proprietary data formats. For example, Bruker MSI platforms collect raw data in the proprietary .d format, which is commonly analyzed using SCiLS Lab, a dedicated software for MSI data analysis. While providing integrated workflows, such proprietary tools limit transparent and flexible data processing.

To facilitate data exchange and vendor-independent analysis, imzML (Schramm *et al*. 2012) has emerged as the standard open format for MSI data. Major MSI vendors now allow the export of raw data to imzML, enabling downstream analysis using a wide range of open-source tools. For example, pyimzML (https://github.com/alexandrovteam/pyimzML) is a parser to access imzML datasets in Python, while Cardinal (Bemis *et al*. 2023) is an R package that takes imzML format as input and offers comprehensive functionalities ranging from spectral preprocessing to statistical and machine learning analyses for MSI, including Spatial Shrunken Centroids (SSC) (Bemis *et al*. 2016) for multivariate segmentation and Dirichlet Gaussian Mixture Modeling (DGMM) (Guo *et al*. 2019) for unsupervised univariate segmentation. In addition, METASPACE (Palmer *et al*. 2017) is a cloud-based platform for metabolite annotation of MSI datasets and hosts a public imzML repository.

Although ion mobility has become increasingly important, the open MSI workflows above do not natively support ion mobility–resolved data. For example, Cardinal (Bemis *et al*. 2023) does not support ion mobility directly. METASPACE (Palmer *et al*. 2017) relies only on m/z for annotation and does not yet leverage the ion mobility information. Proprietary software such as SCiLS (Bruker) and HDI (Waters) excludes ion mobility information when exporting imzML, as there is no standard format to store ion mobility in imzML.

In parallel, existing open-source tools for ion mobility-coupled mass spectrometry are primarily designed for non-imaging workflows. For example, AlphaTims (Willems *et al*. 2021) and OpenTIMS (Lacki *et al*. 2021) enable parsing raw timsTOF spectra and provide access to high-dimensional MS data, but pixel coordinates are omitted. TIMSCONVERT (Luu *et al*. 2022) supports conversion of raw timsTOF imaging data into imzML, but lacks spectral processing. DEIMoS (Colby *et al*. 2022) provides ion mobility processing functionalities such as CCS calibration, but operates on the non-imaging mzML format.

Recently, several tools specifically designed for ion mobility MSI have been developed. Wang *et al.* presented prepIMS (Wang *et al*. 2026), a preprocessing workflow for MALDI-TIMS imaging data that enables detection of isobaric ion signals. However, prepIMS primarily focuses on peak processing, and downstream applications with IM-resolved features are less covered. Tortorella *et al.* developed Pyxis (Tortorella *et al*. 2025), which integrates raw data processing, visualization, statistical analysis and identification for ion mobility MSI data from multiple vendors. However, it is a commercial software with limited free access.

Importantly, none of these tools export the data into the standard imzML format that incorporates ion mobility information, and therefore are not directly compatible with the diverse set of tools that already exist for downstream statistical and machine learning analyses of MSI.

## 3 Methods

**Figure 1:**
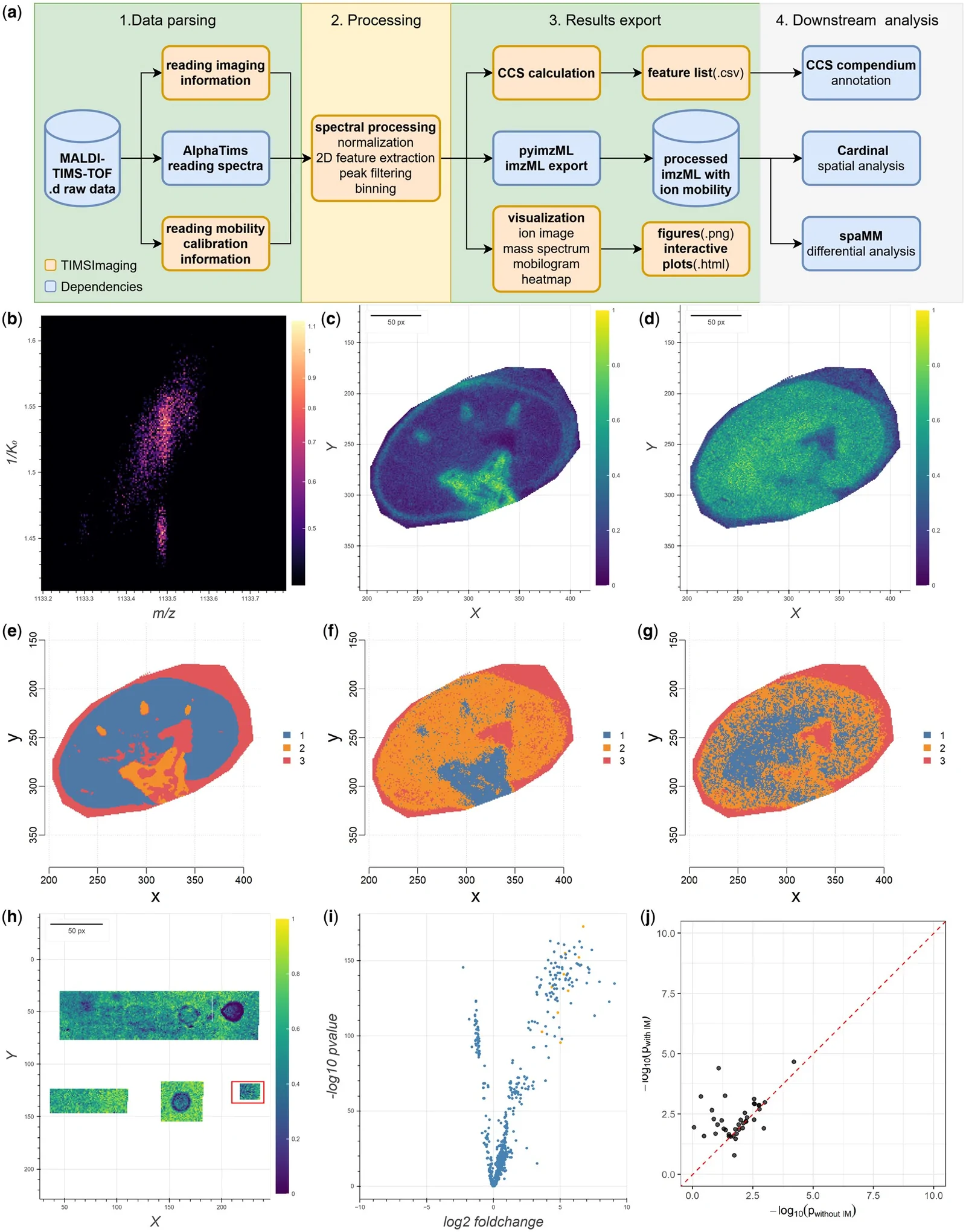
(a) Overview of TIMSImaging. (b) 2D intensity heatmap of two ions with the same m/z 1133.471 and different ion mobility, which can not be resolved in the 1D mass spectrum, but 2D feature extraction separates them. (c) The ion image of (m/z 1133.4715, 1/$K_{0}$ 1.459). (d) The ion image of (m/z 1133.4876, 1/$K_{0}$ 1.531). (e) Multivariate Spatial Shrunken Centroids segmentation of the mouse kidney dataset (f)-(g) Univariate DGMM segmentation on the two isobaric ions. (h) Region definition of the natural products dataset. The red box is the matrix/media region, while the other pixels are the culture region. (i) volcano plot of Wilcoxon rank-sum differential analysis, each point is an ion feature, those at top right present higher intensity in the culture region than in the matrix/media region with high significance scores, orange points are precursors selected by Shepherd *et al*. using SCiLS Lab. (j) comparison of *P*-values from spatial mixed effects modeling. X axis is negative log *P*-value from processing without ion mobility, Y axis is negative log *P*-value from corresponding IM-resolved features.

Figure 1a presents an overview of the proposed workflow, which provides access to raw data with the ion mobility dimension, enables IM-aware processing and exports imzML with ion mobility for downstream analysis. The workflow comprises four stages:

1. *Raw data parsing* TIMSimaging parses raw data (.d format) from Bruker timsTOF instruments, it relies on AlphaTims (Willems *et al*. 2021) to access high-dimensional spectra, and implements custom functions to extract spatial coordinates and ion mobility calibration metadata.
2. *Spectral processing* The data undergo optional region of interest (ROI) selection and total ion current (TIC) normalization, and a mean spectrum is computed. A two-dimensional feature extraction algorithm is then applied to obtain a feature list with m/z, ion mobility, and intensity. All spectra are subsequently binned with respect to the features.
3. *Result export* The results can be exported in multiple formats. CCS values are derived from the peak list using a linear calibration. Centroided spectra are exported in imzML format, with ion mobility stored as an extra array. In addition, the processed data are rendered into multiple visualization forms, including ion image, mass spectrum, mobilogram and intensity heatmap, as well as in an interactive GUI that links these components.
4. *Downstream analysis* The processed data can serve as input to the broader open-source MS ecosystem, facilitating different downstream analysis, including molecular annotation via CCS compendium matching, statistical and machine learning analyses using Cardinal and other tools.

We briefly discuss the details of the methods below.

### 3.1 Spectral processing

Spectral processing aims to extract ion peaks and generate a feature list with m/z and ion mobility columns, each feature corresponds to a channel of the hyperspectral image. TIMSImaging formulates the problem of feature extraction as follows. It treats a 2D spectrogram as a point set in (m/z, $1/K_{0}$) metric space, and defines a peak as a subset of the points such that (1) it is represented by the point with the highest intensity, and (2) its points are close together and disjoint from other subsets.

The feature extraction algorithm consists of two steps shown in Supplemental Fig. S1, available as supplementary data at *Bioinformatics* online. Step 1 aggregates the points into subsets. TIMSImaging creates a graph where connectivity is parameterized by an intensity threshold, then traverses the graph to group points into connected components (subsets). Two points are connected if (1) both of them have intensities higher than the threshold, and (2) their m/z and mobility differences are no more than user-defined tolerances. Step 2 finds local maxima within each subset. Each subset is converted to a dense matrix, an adaptive scanning window is applied to find local maxima. If multiple local maxima are detected, the subset is divided further. The m/z and $1/K_{0}$ values of a peak are recorded as intensity-weighted average over the subset, and the intensities are summed. Features can be optionally filtered by point count and total intensity. Finally, all spectra are centroided based on the features, forming an N×L intensity array, where *N* is the number of pixels and *L* is the number of features.

### 3.2 CCS calculation

CCS calculation aims to compute CCS values from $1/K_{0}$ ion mobility values, according to reference ion mobility values of the calibrants. Currently TIMSImaging supports a subset of Agilent Tune Mix Ions (Stow *et al*. 2017) as calibrants. The calibration information is automatically parsed from the metadata, then a linear model (see Supplemental Section 2.2, available as supplementary data at *Bioinformatics* online) is fit to transform $1/K_{0}$ values of the feature list into CCS values.

### 3.3 imzML export with mobility

The N×L intensity array obtained from spectral processing and pixel coordinates are written into imzML format using the custom pyimzML from TIMSCONVERT (https://github.com/gtluu/pyimzML). The continuous, centroided imzML contains a common ion moblity domain and is compatible with spatial analysis in Cardinal as well as differential abundance analysis.

### 3.4 Implementation details

TIMSImaging is implemented in Python and is tested on Windows and Linux. Its full functionality to access the raw data is limited on MacOS due to the fact that Bruker dynamic library is currently not available on Mac. The interactive visualization is embedded in Jupyter Notebooks (Supplemental Fig. S2, available as supplementary data at *Bioinformatics* online). A sparse graph representation and Numba JIT compiler are used to accelerate large data processing.

## 4 Case studies

We illustrate TIMSImaging in three case studies with different analyte types, sample types, and downstream tasks. Additional details can be found in Supplemental Section 3, available as supplementary data at *Bioinformatics* online. All the case studies are documented in interactive HTML Vignettes generated by Quarto. We also provide separate reproducible Jupyter Notebook and R Markdown files for ease of use.

### 4.1 Case study 1: segmentation of peptides images on mouse kidney tissue

#### 4.1.1 Overview

Li *et al.* studied spatial proteomics of mouse kidney tissue (Li *et al*. 2025). This case study uses the MALDI-TIMS-MS1 data to showcase the integration of processing with TIMSImaging and image segmentation with Cardinal, and highlight the benefits of IM-aware processing.

#### 4.1.2 TIMSImaging separated isomeric and isobaric features by ion mobility

Isomeric ions have the exact same chemical formula but different structure, while isobaric ions present similar m/z but different formulas, in both cases it is challenging to separate features solely by m/z, but ion mobility provides an orthogonal separation dimension. For example, two features were detected with very close m/z but different ion mobility values, (m/z 1133.4715, $1/K_{0}$ 1.459) and (m/z 1133.4876, $1/K_{0}$ 1.531), respectively (Fig. 1b). The features showed different spatial distributions (Fig. 1c and d). More examples are in Supplemental Fig. S3, available as supplementary data at *Bioinformatics* online. The IM-aware feature detection helped find informative ions among the isobaric background.

#### 4.1.3 IM-aware spectral processing reduced noise of single ion image segmentation

Spatial Shrunken Centroids (SSC) (Bemis *et al*. 2016) segmentation was first applied to the IM-aware processed data, resulting in 3 segments on the tissue, namely background, cortex, and medulla (Fig. 1e). The result was consistent with segmentation using SCiLS Lab (Supplemental Fig. S4, available as supplementary data at *Bioinformatics* online).

To further illustrate the role of ion mobility and compatibility of TIMSImaging with diverse types of downstream analyses, spatial Dirichlet Gaussian Mixture Modeling (DGMM) in Cardinal (Guo *et al*. 2019) was applied to individual features. Two isobaric features (m/z 1133.471) with different ion mobilities were selected. DGMM segmentation was performed separately on each of them (Fig. 1f, g), and on the pixel-wise summed image (Supplemental Fig. S5, available as supplementary data at *Bioinformatics* online). The segments from one isobar ($1/K_{0}=1.459$) showed clearer boundaries compared with the other isobar as well as the summed feature, indicating that IM-resolved feature can reduce ambiguity in the segmentation results.

### 4.2 Case study 2: differential analysis of natural products images on microbial cultures

#### 4.2.1 Overview

Shepherd *et al.* studied MALDI-TIMS-MS2 imaging and annotation of natural products in fungal-bacterial co-culture (Shepherd *et al*. 2025). The authors first conducted a MALDI-TIMS-MS1 experiment, then selected precursors associated with microbial culture regions as targets for iprm-PASEF MS2 imaging acquisition. The case study showcases the compatibility of TIMSImaging with differential analysis using various general-purpose methods, and its potential to facilitate experiment design.

#### 4.2.2 Differential analysis on the IM-resolved features found ions presenting a differential abundance between ROIs

Differential analysis aimed to find features with systematically higher intensity in the microbial culture region than in the media/matrix region (Fig. 1h). First we performed the Wilcoxon’s rank sum test on the IM-resolved features to produce comparable results in the original publication. We used the same setting as the original publication, which treated the three microbial regions as group A, and the media/matrix region as group B. The result (Fig. 1i) included 9 precursors selected in the original publication presented high fold change and significance, including previously reported coproporphyrin III (m/z 655.2736, $1/K_{0}$ 1.272) (Supplemental Fig. S6b, available as supplementary data at *Bioinformatics* online). However, the Wilcoxon’s rank sum test is limited in that (1) it defines 3 heterogeneous microbial culture regions as one group; and (2) it treats spatially correlated image pixels as independent.

To overcome these limitations, and to demonstrate the compatibility of the proposed processing with multiple statistical models, we fit a linear mixed effects spatial model with Matérn random effect from spatial transcriptomics (Ospina *et al*. 2024) (Supplemental Section 3.2.3, available as supplementary data at *Bioinformatics* online). We considered a subset of ROIs with the *G. arilaitensis* region as group A, and the media/matrix region as group B. We found that while the *P*-values from non-spatial modeling were exceedingly small, the *P*-values from the spatial modeling were more realistic (Supplemental Fig. S8, available as supplementary data at *Bioinformatics* online).

We further compared the spatial modeling results between the features obtained with and without IM-aware processing (Fig. 1j). The processing without ion mobility was performed by TIMSCONVERT and Cardinal (Supplemental Section 3.2.4, available as supplementary data at *Bioinformatics* online), then matched with TIMSImaging features according to their m/z. The processing by TIMSImaging produced smaller *P*-values, suggesting that ion mobility improved the signal-to-noise ratio and sensitivity of differential analysis.

### 4.3 Case study 3: lipids annotation of mouse skin tissue

#### 4.3.1 Overview

Cryopreserved mouse skin was analyzed using MALDI-TIMS-MS1 imaging to identify the lipid composition in different skin compartments. This case study showcases the value of IM-derived CCS for annotating lipids with a public CCS database.

#### 4.3.1 IM-resolved feature detection enabled CCS-based annotation

The feature list was annotated with the CCS Compendium database (Picache *et al*. 2019) (Supplemental Section 3.3, available as supplementary data at *Bioinformatics* online), 77 features had at least one putative annotation and the top results are shown in Supplemental Table S3, available as supplementary data at *Bioinformatics* online. Among features with multiple hits, CCS helped reduce ambiguous annotations. For example, two features (m/z 766.5358 CCS = 283.1 ${Å}^{2}$ and m/z 766.5354 CCS = 287.8 ${Å}^{2}$) are annotated as two different isomers of PE (36:02), and present different distributions on the tissue (Supplemental Fig. S10, available as supplementary data at *Bioinformatics* online).

Furthermore, we exported the processed data and annotated the imzML using METASPACE (Palmer *et al*. 2017) with and without ion mobility and CCS-based annotation (Supplemental Fig. S11, available as supplementary data at *Bioinformatics* online). 37 features were annotated by both methods, supported by both spatial confidence from METASPACE FDR control and structural confidence from CCS. This suggests that CCS-based annotation can be a complementary approach to the current standard MSI annotation workflow.

## 5 Discussion

TIMSImaging provides an open-source approach to process and visualize MALDI-TIMS-MSI data from timsTOF instruments. The implemented feature detection algorithm demonstrates the ability to separate isobaric signals by ion mobility on datasets of diverse sample and analyte types, and IM-resolved features improve downstream analyses in both spatial and spectral domain. Together, these capabilities extend existing MSI workflows for data exploration and interpretation.

Nevertheless, several limitations remain. First, TIMSImaging currently supports only single sample, MS1-level imaging data from timsTOF. Molecular identification based solely on MS1 data remains putative, processing steps such as deisotoping and quality control are not implemented. Second, although ion mobility enables separation of isomeric and isobaric signals, the complex nature of the samples prevents definitive confirmation of isomeric species when multiple features are detected at the same m/z in the sample, more work is needed to confirm whether they are different isomers or the conformers of the same identity. Finally, certain usability issues persist, for example integration with existing R-based MSI workflows requires users to operate across both Python and R environments. Future development will focus on addressing these limitations, including integrating the processing functionalities with Cardinal, adding support for multi-sample and MS2-level imaging datasets, and refining user interface to streamline cross-platform workflows.

## Supplementary Material

btag624_Supplementary_Data

## Data Availability

All datasets used in the case studies are public. The mouse kidney peptides dataset is available at MassIVE, with ID MSV000096373 (https://doi.org/10.25345/C54Q7R252). The microbial natural products dataset is available at MassIVE, with ID MSV000097837 (https://doi.org/10.25345/C5JH3DF50). The mouse skin lipids dataset is available at https://zenodo.org/records/18713016.

## References

[btag624-B1] Alexandrov T. Spatial metabolomics and imaging mass spectrometry in the age of artificial intelligence. Annu Rev Biomed Data Sci 2020;3:61–87. 34056560 10.1146/annurev-biodatasci-011420-031537 PMC7610844

[btag624-B2] Bemis KA , Föll MC, Guo D et al Cardinal v.3: a versatile open-source software for mass spectrometry imaging analysis. Nat Methods 2023;20:1883–6. 37996752 10.1038/s41592-023-02070-z

[btag624-B3] Bemis KD , Harry A, Eberlin LS et al Probabilistic segmentation of mass spectrometry (MS) images helps select important ions and characterize confidence in the resulting segments. Mol Cell Proteomics 2016;15:1761–72. 26796117 10.1074/mcp.O115.053918 PMC4858953

[btag624-B4] Colby SM , Chang CH, Bade JL et al DEIMoS: an open-source tool for processing high-dimensional mass spectrometry data. Anal Chem 2022;94:6130–8. 35430813 10.1021/acs.analchem.1c05017 PMC9047447

[btag624-B5] Djambazova KV , Klein DR, Migas LG et al Resolving the complexity of spatial lipidomics using MALDI TIMS imaging mass spectrometry. Anal Chem 2020;92:13290–7. 32808523 10.1021/acs.analchem.0c02520

[btag624-B6] George AC , Schmitz I, Rouvière F et al Interplatform comparison between three ion mobility techniques for human plasma lipid collision cross sections. Anal Chim Acta 2024;1304:342535. 38637036 10.1016/j.aca.2024.342535

[btag624-B7] Giles K , Ujma J, Wildgoose J et al A cyclic ion mobility-mass spectrometry system. Anal Chem 2019;91:8564–73. 31141659 10.1021/acs.analchem.9b01838

[btag624-B8] Guo D , Bemis K, Rawlins C et al Unsupervised segmentation of mass spectrometric ion images characterizes morphology of tissues. Bioinformatics 2019;35:i208–17. 31510675 10.1093/bioinformatics/btz345 PMC6612871

[btag624-B9] Lacki MK et al OpenTIMS. TimsPy, and TimsR: open and easy access to timstof raw data. J Proteome Res 2021;20:2122–9. 33724840 10.1021/acs.jproteome.0c00962

[btag624-B10] Leontyev D , Olivos H, Shrestha B et al Desorption electrospray ionization cyclic ion mobility-mass spectrometry imaging for traumatic brain injury spatial metabolomics. Anal Chem 2024;96:13598–606. 39106040 10.1021/acs.analchem.4c02394 PMC11339727

[btag624-B11] Li MJ , Meyer LC, Meier N et al Spatial proteomics by parallel accumulation-serial fragmentation supported MALDI MS/MS imaging: a first glance into multiplexed and spatial peptide identification. Rapid Commun Mass Spectrom 2025;39:e10006. 39910729 10.1002/rcm.10006 PMC11799399

[btag624-B12] Liu M , Zhang C, Xu L et al Ion mobility-mass spectrometry imaging: advances in biomedical research. BioTech (Basel) 2025;14:98. 41440177 10.3390/biotech14040098 PMC12730489

[btag624-B13] Luu GT , Freitas MA, Lizama-Chamu I et al TIMSCONVERT: a workflow to convert trapped ion mobility data to open data formats. Bioinformatics 2022;38:4046–7. 35758608 10.1093/bioinformatics/btac419 PMC9991885

[btag624-B14] Ma X , Fernandez FM. Advances in mass spectrometry imaging for spatial cancer metabolomics. Mass Spectrom Rev 2024;43:235–68. 36065601 10.1002/mas.21804 PMC9986357

[btag624-B15] Ngai YT , Briggs MT, Mittal P et al Mass spectrometry imaging protocol for spatial mapping of lipids, N-glycans and peptides in murine lung tissue. Rapid Commun Mass Spectrom 2024;38:e9721. 38525810 10.1002/rcm.9721

[btag624-B16] Ospina OE , Soupir AC, Manjarres-Betancur R et al Differential gene expression analysis of spatial transcriptomic experiments using spatial mixed models. Sci Rep 2024;14:10967. 38744956 10.1038/s41598-024-61758-0 PMC11094014

[btag624-B17] Paglia G , Smith AJ, Astarita G. Ion mobility mass spectrometry in the omics era: challenges and opportunities for metabolomics and lipidomics. Mass Spectrom Rev 2022;41:722–65. 33522625 10.1002/mas.21686

[btag624-B18] Palmer A , Phapale P, Chernyavsky I et al FDR-controlled metabolite annotation for high-resolution imaging mass spectrometry. Nat Methods 2017;14:57–60. 27842059 10.1038/nmeth.4072

[btag624-B19] Picache JA , Rose BS, Balinski A et al Collision cross section compendium to annotate and predict multi-omic compound identities. Chem Sci 2019;10:983–93. 30774892 10.1039/c8sc04396e PMC6349024

[btag624-B20] Schramm T , Hester Z, Klinkert I et al imzML–a common data format for the flexible exchange and processing of mass spectrometry imaging data. J Proteomics 2012;75:5106–10. 22842151 10.1016/j.jprot.2012.07.026

[btag624-B21] Shepherd RA , Luu GT, Sanchez LM. MALDI-TIMS-MS(2) imaging and annotation of natural products in fungal-bacterial coculture. Anal Chem 2025;97:18867–72. 40864852 10.1021/acs.analchem.5c02787 PMC12413305

[btag624-B22] Spraggins JM , Djambazova KV, Rivera ES et al High-performance molecular imaging with MALDI trapped ion-mobility time-of-flight (timsTOF) mass spectrometry. Anal Chem 2019;91:14552–60. 31593446 10.1021/acs.analchem.9b03612 PMC7382025

[btag624-B23] Stow SM , Causon TJ, Zheng X et al An interlaboratory evaluation of drift tube ion mobility-mass spectrometry collision cross section measurements. Anal Chem 2017;89:9048–55. 28763190 10.1021/acs.analchem.7b01729 PMC5744684

[btag624-B24] Tortorella S , Bessler S, Arturi G et al Advancing spatial-omics through Pyxis, vendor-neutral software for ion mobility mass spectrometry imaging data analysis. Anal Bioanal Chem 2025. 10.1007/s00216-025-06281-5 PMC13619667 41420743

[btag624-B25] Wang L , Xie C, Guo L et al prepIMS: a robust data preprocessing workflow for ion mobility mass spectrometry imaging. Anal Chim Acta 2026;1384:344951. 41513366 10.1016/j.aca.2025.344951

[btag624-B26] Willems S , Voytik E, Skowronek P et al AlphaTims: indexing trapped ion mobility spectrometry-tof data for fast and easy accession and visualization. Mol Cell Proteomics 2021;20:100149. 34543758 10.1016/j.mcpro.2021.100149 PMC8526765

[btag624-B27] Zhang H , Lu KH, Ebbini M et al Mass spectrometry imaging for spatially resolved multi-omics molecular mapping. Npj Imaging 2024;2:20. 39036554 10.1038/s44303-024-00025-3 PMC11254763

