# Supplementary material for: TIMSImaging: an open and interoperable workflow for trapped ion mobility mass spectrometry imaging data processing and visualization
Source: Bioinformatics. 2026 Aug 25;42(9):btag624. doi: 10.1093/bioinformatics/btag624 (PMC13537506; doi:10.1093/bioinformatics/btag624)
Supplement: Supplementary file 1 [file btag624_supplementary_data.pdf]

Supplmentary information for *TIMSImaging: An open and interoperable workflow for trapped ion mobility mass spectrometry imaging data processing and visualization*

Yinyue Zhu[1], Kylie Bemis[1], Sai Srikanth Lakkimsetty[1], Mujia Jenny Li[2,4], Larissa Chiara Meyer[2,3], Andreas Weber-Steinhilber[2], Melanie Christine Föll[2], and Olga Vitek[1, *]

[1]Khoury College of Computer Sciences, Northeastern University, Boston, MA, United States
[2]Institute of Surgical Pathology, University of Freiburg, Faculty of Medicine, Freiburg, Germany
[3]Faculty of Biology, University of Freiburg, Freiburg, Germany
[4]Institute for Pharmaceutical Sciences, University of Freiburg, Freiburg, Germany

∗**Corresponding author:** o.vitek@northeastern.edu

1 Background

Table S1: Summary of computational tools for mass spectrometry imaging and ion mobility data analysis.

Name	Description	Ion mobility compatibility	MSI dedicated	Accessibility	Language
pyimzML	Parser and writer for imzML	No	Yes	Open source	Python
Cardinal	Statistical and machine learning analysis for MSI	No	Yes	Open source	R
METASPACE	Metabolites/lipids annotation for MSI	No	Yes	Free web service with quota	Python
AlphaTims	Programmatic access to raw TIMS data	Yes	No	Open source	Python
OpenTIMS	Programmatic access to raw TIMS data	Yes	No	Open source	C++, Python, R
TIMSCONVERT	Format converter for raw TIMS data	Yes	No	Open source	Python
DEIMoS	Spectral processing for multi-dimensional mzML	Yes	No	Open source	Python
prepIMS	Spectral processing for raw TIMS-MSI data	Yes	Yes	Open source	Python
Pyxis	Integrated analysis for MSI	Yes	Yes	Commercial	—
SCiLS Lab	Integrated analysis for MSI	Yes	Yes	Commercial	—

2 Methods

2.1 2D feature extraction algorithm

An overview of the feature extraction algorithm in TIMSImaging is shown in **FigureS1**. The algorithm is performed in a discrete "index space", where both m/z and $1/K_0$ (inverse reduced ion mobility) are represented as integer indices instead of actual values. Points are aggregated into subsets by proximity, followed by local maxima detection within each subset, and finally indices of peaks are mapped to actual values.

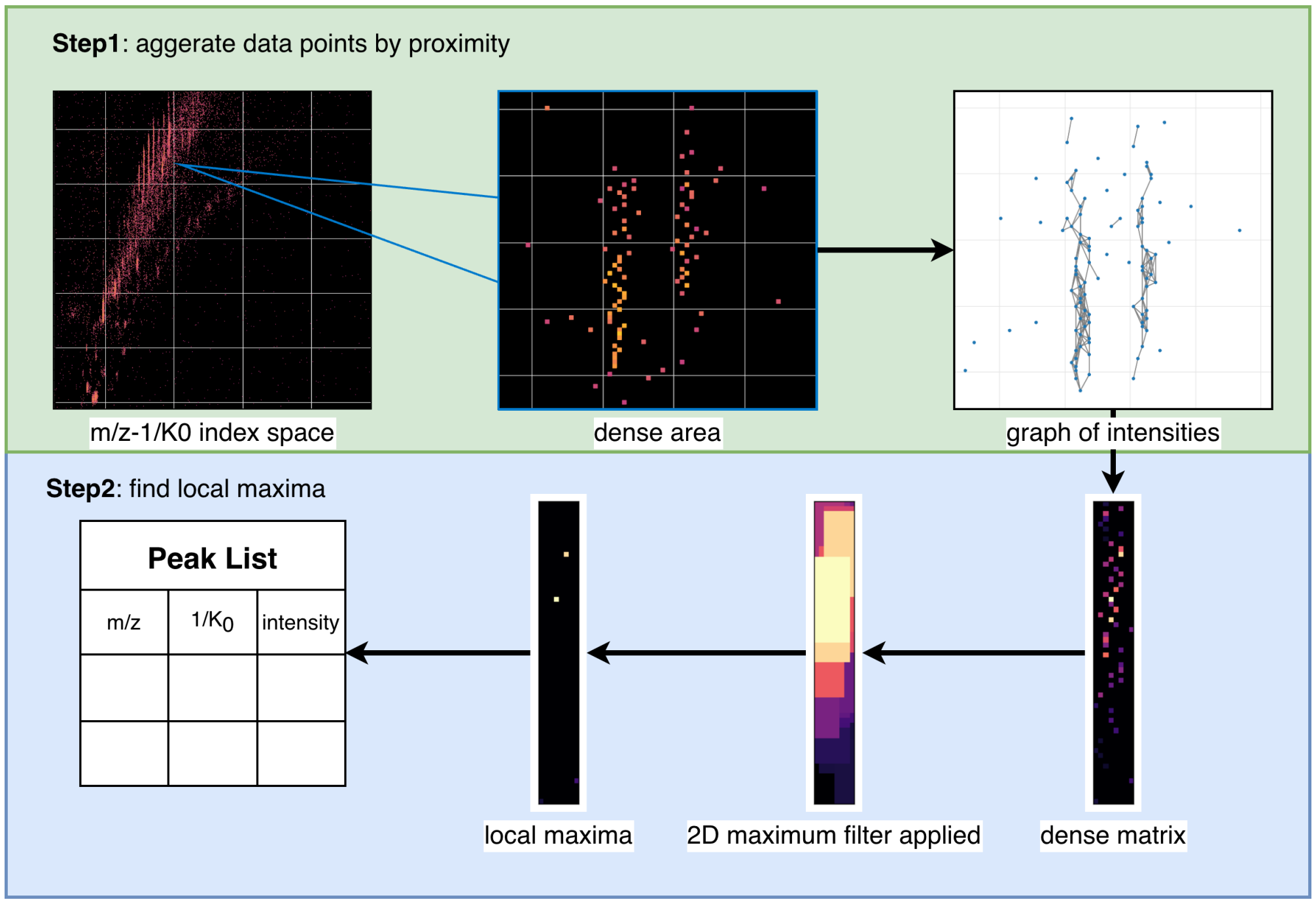

Figure S1: **An overview of 2D feature detection algorithm in TIMSImaging.** The algorithm locates dense areas in two-dimensional m/z and ion mobility space by building a radius nearest neighbors graph (Step 1), then detects local maxima within each area (Step 2).

There are two reasons to perform feature detection in the underlying index space. First, in the value space m/z and ion mobility are in different units and scales, and the distance metric is not well defined. Second, the raw data are stored as indices, which need closed functions from the Bruker's TDFSDK dynamic library for conversion to values, specifically `tims_index_to_mz` for m/z, and `tims_scannum_to_oneoverk0` for ion mobility. However, the SDK is only available on Windows and Linux system. By detecting peaks in the index space, the algorithm works even if the SDK is not available.

2.2 CCS calculation

According to the Mason-Shamp equation[1]

$$K = \frac{3q}{16N}\sqrt{\frac{2\pi}{\mu k_B T}}\frac{1}{\Omega}$$

Symbol	Quantity	Units
K	Ion mobility	$cm^2\ V^{-1}\ s^{-1}$
q	Ion charge	C
N	Number density of buffer gas	m^{-3}
μ	Reduced mass, $\frac{m_{\text{ion}} \cdot m_{\text{gas}}}{m_{\text{ion}} + m_{\text{gas}}}$	kg
k_B	Boltzmann constant	$J \cdot K^{-1}$
T	Temperature	K
Ω	Collision cross-section (CCS)	m^2 (or $\mathring{A}^2$)

There is a relationship:

$$\Omega \propto \frac{1}{K_0}\frac{1}{\sqrt{\mu}}$$

where $1/K_0$ is the inverse reduced ion mobility, which is normalized by temperature and pressure. The ion mobility information is recorded as $1/K_0$ in timsTOF data. TIMSImaging fits a linear model

$$\Omega = a \cdot \frac{1}{K_0}\frac{1}{\sqrt{\mu}} + b$$

where $\frac{1}{K_0}$ is the measured ion mobility values of calibrants recorded in metadata, μ is calculated from formulas of the calibrants, and Ω are reference CCS values from literature[1]. a and b are parameters.

2.3 Visualization

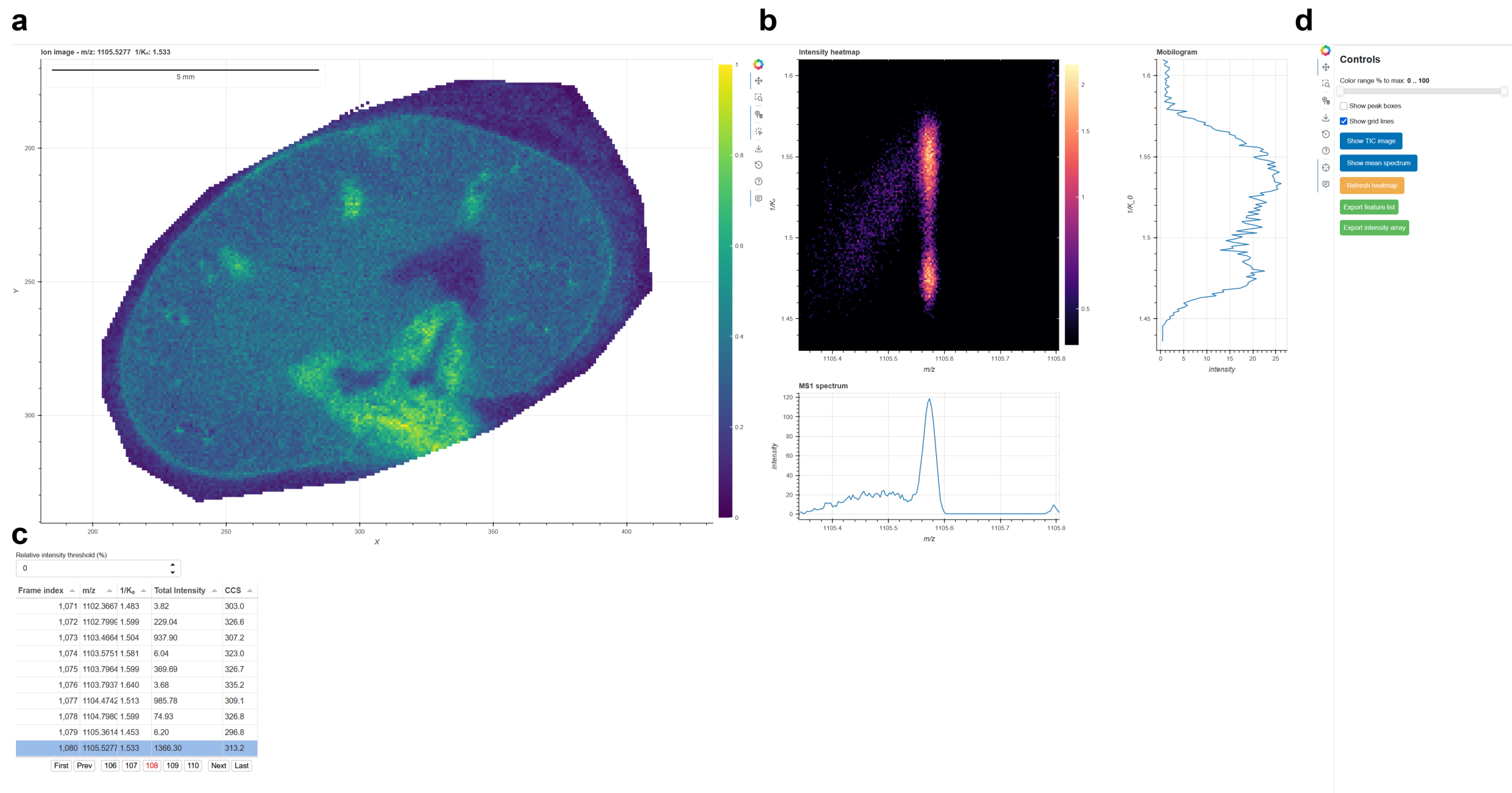

Figure S2: A screenshot from the TIMSImaging GUI. By clicking on pixels in the image (top left), users can view its mass spectrum, mobilogram and 2D heatmap combining these two (middle right). All the plots support interactivity such as dragging, zooming, hovering, and download. Also, users can click entries in the peak list (bottom left) to investigate ion image and spectral details of that feature. The menu (right) provides summarizing functions such as showing TIC image and mean spectrum, visual adjustment, as well as file export options.

3 Case studies

3.1 Case study 1: Segmentation on peptides imaging of mouse kidney tissue

3.1.1 Experimental details

The MALDI-TIMS-MS1 imaging dataset of tryptic peptides on wildtype mouse kidney tissue was acquired in the positive mode using a timsTOF fleX mass spectrometer (Bruker Daltonics, Bremen, Germany) at a pixel size of 50×50 μm, the range of m/z was set to 800-2,000, the range of $1/K_0$ to 1.2-2.1 $\text{V} \cdot \text{s/cm}^2$. The data contains 38267 pixels in total and is over 11 GB in raw(.d) format.

3.1.2 Data processing with TIMSImaging

Processing was performed on an ROI of kidney tissue with 22978 pixels, using parameters described in Vignette 1. 3110 features were detected, including all 5 precursors reported in the literature, with no more than 10 ppm in m/z and 0.02 V· s/cm2 in $1/K_0$. The processed data were exported in continuous centroided imzML format of 1.06 GB, with ion mobility arrays, then loaded into Cardinal for downstream segmentation.

The following figures show results on the mouse kidney peptides dataset. Figure S3 shows separation of isomeric features by ion mobility. Figure S4 shows segmentation by SCiLS Lab. Figure S5 shows univariate segmentation ignoring ion mobility separation and is subject to comparison with Figure1 f-g in the main text.

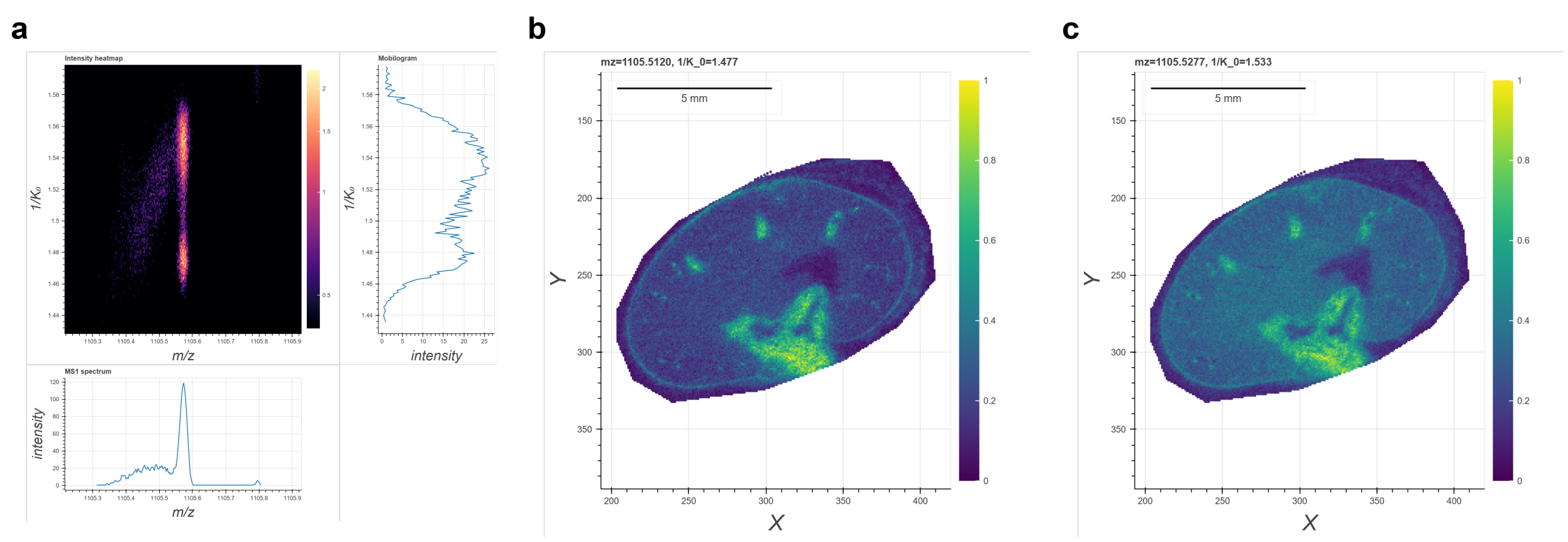

Figure S3: **Mouse kidney peptides dataset:** example isomeric/isobaric ion images. (a) TIMSImaging detected two peaks in the $m/z - 1/K_0$ intensity heatmap, which cannot be distinguished by 1D mass spectrum, but can be separated by ion mobility. (b) image of ion (m/z 1105.5120, $1/K_0$ 1.477). (c) image of ion (m/z 1105.5277, $1/K_0$ 1.533). Though TIMSImaging reported slightly different m/z values due to weighted average summarization of m/z, the apexes of the two features locate at exactly the same m/z position.

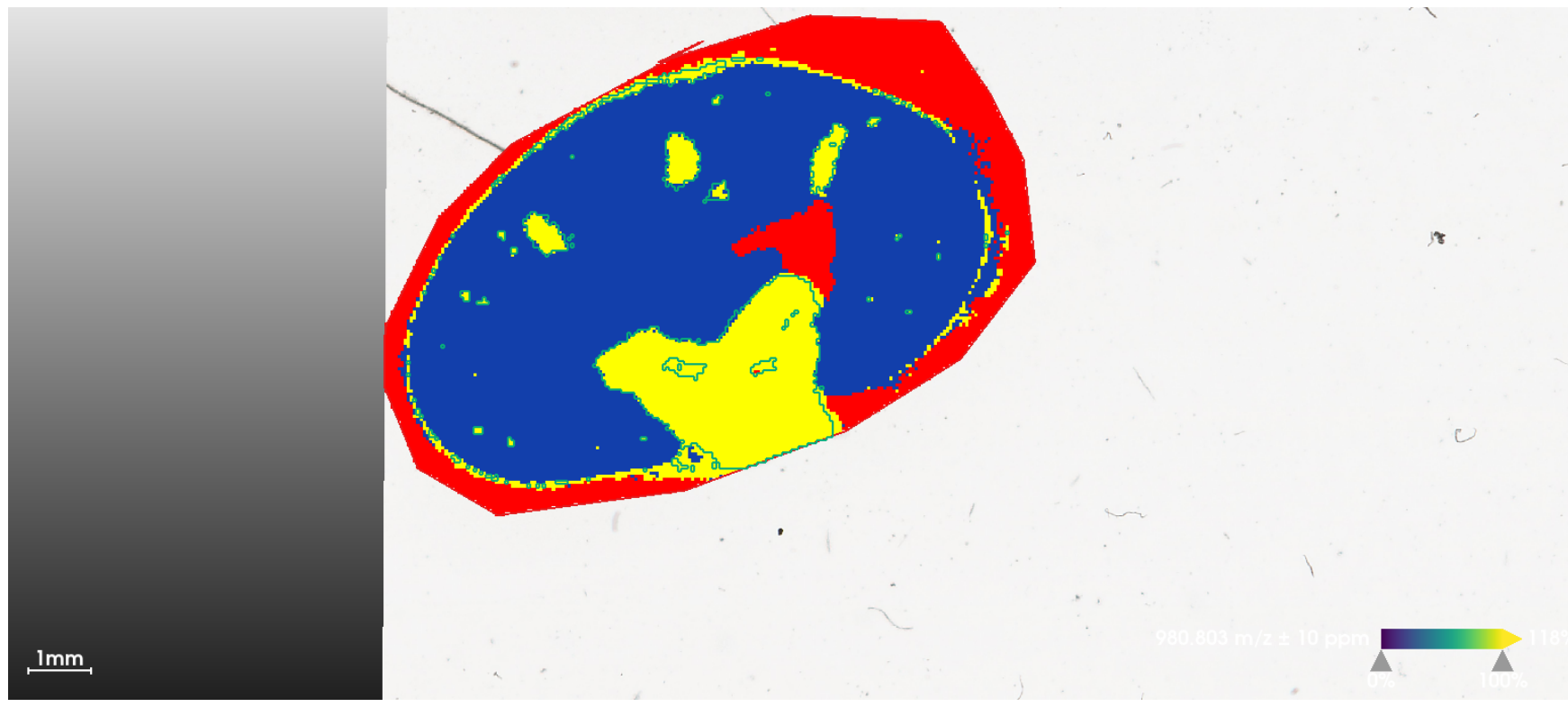

Figure S4: **Mouse kidney peptides dataset:** multivariate bisecting K-means segmentation result by SCiLS Lab.

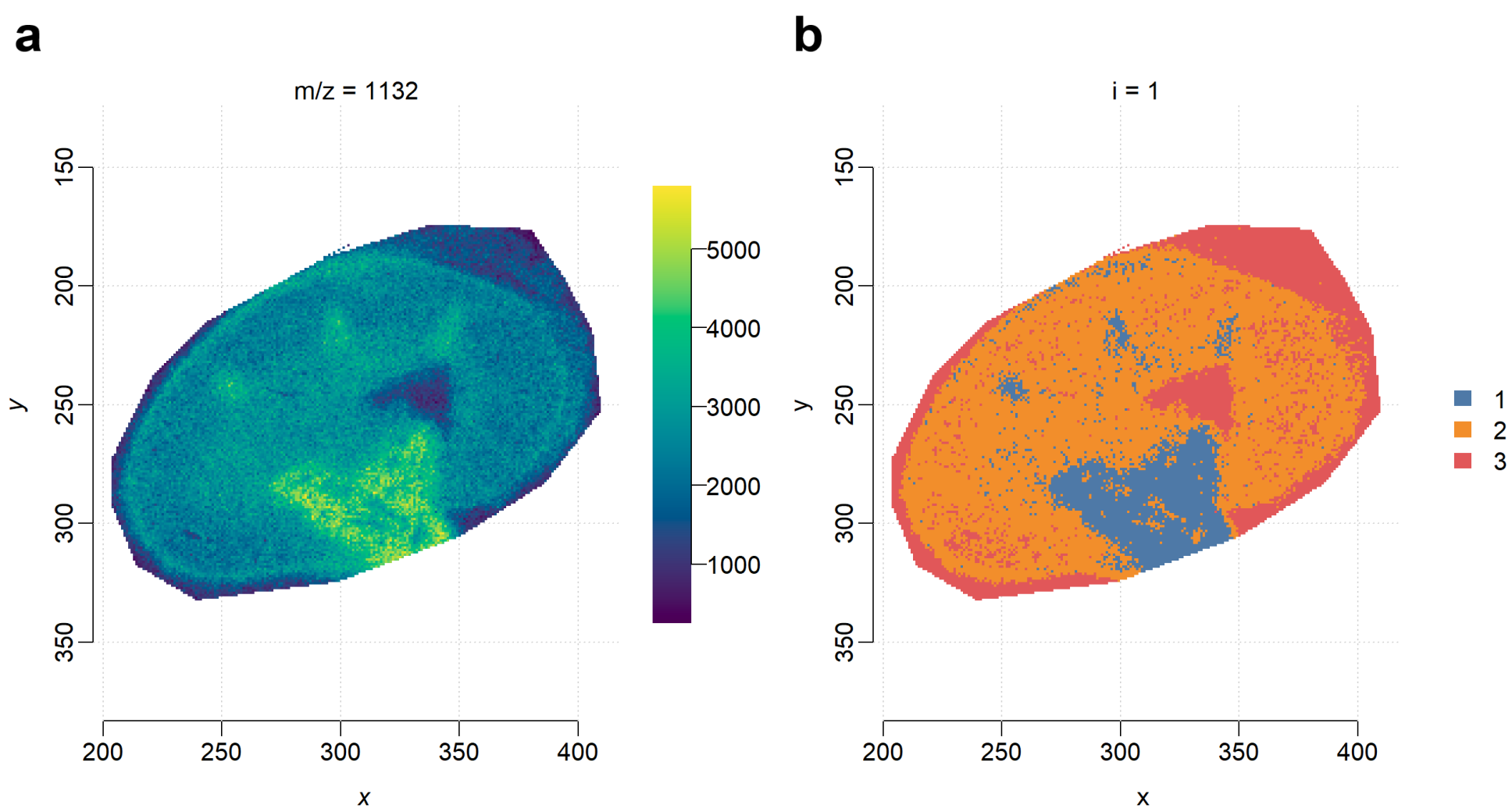

Figure S5: **Mouse kidney peptides dataset:** (a) Overlay (pixel-wise sum) of two isobaric ion images at m/z 1132.471 (b) SpatialDGMM segmentation on the summed image.

3.2 Case study 2: Differential analysis of natural products images of microbial cultures

3.2.1 Experimental details

The data was collected by a timsTOF fleX mass spectrometer (Bruker Daltonics) in positive ion mode from m/z 100-1100, and 1/K0 0.4-1.80 Vs/cm2. The dataset consists of four regions: *P. solitum*, *G. arilaitensis*, their co-culture and the media/matrix (Main Text Figure 1h), with 12173 pixels in total. The raw MALDI-TIMS-MS1 data is of 7.3 GB in .d format.

3.2.2 Data processing with TIMSImaging

The data was processed using parameters described in Vignette 2, resulting in 784 ion features closely matching the original report. For example, the authors reported coproporphyrin III ([M+H]+, theoretical m/z 655.2768) were observed at m/z 655.2726, $1/K_0$ 1.2755 ± 0.02 V·s/cm^2, while TIMSImaging detected the feature at 655.2736, $1/K_0$ 1.272 V·s/cm^2, the value reported by TIMSImaging was slightly closer to the theoretical m/z, and the ion mobility difference is within the mobilogram range. The processed data was then exported as an imzML file of 145 MB with ion mobility information.

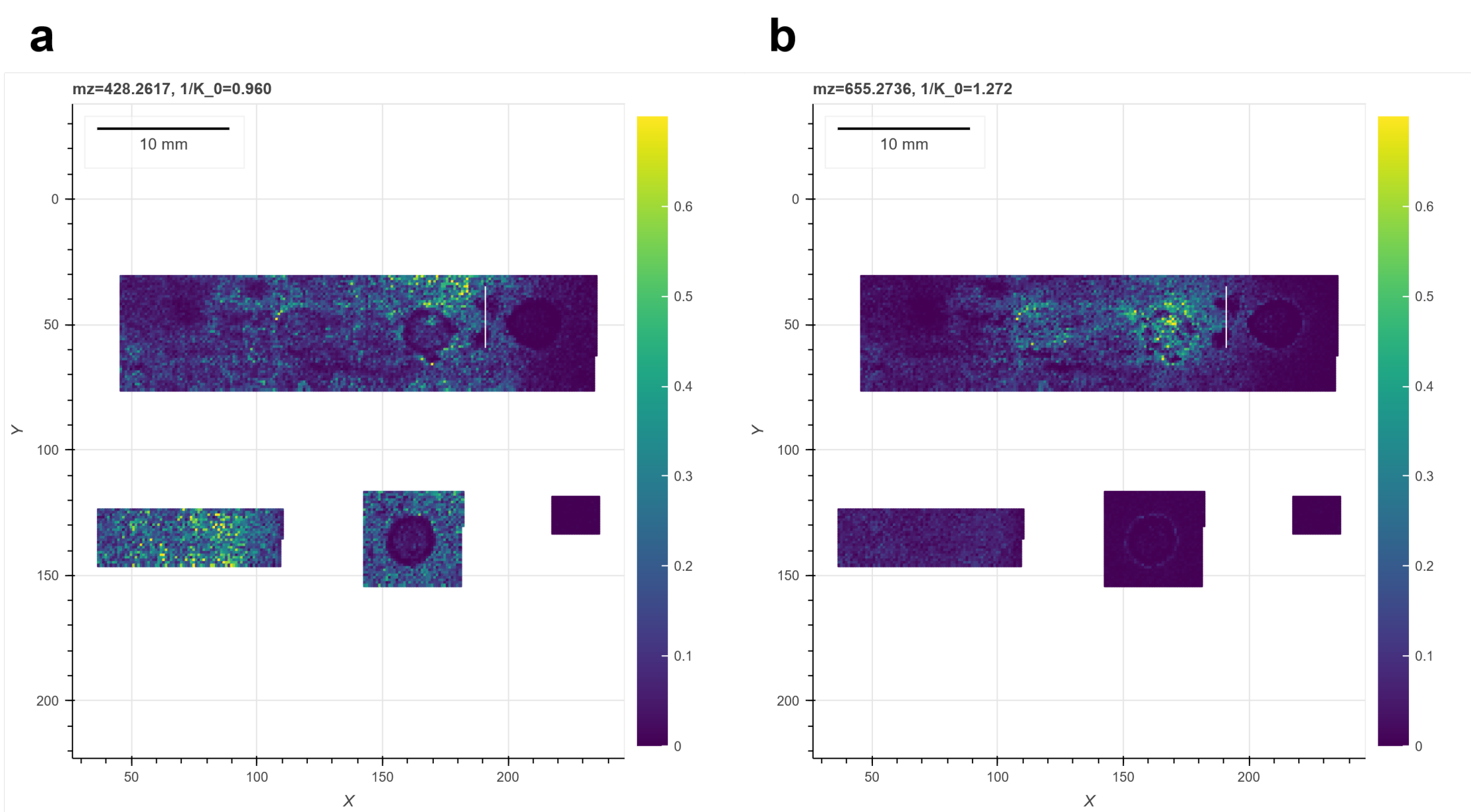

Figure S6: **Microbial natural products dataset:** example differential ion images by Wilcoxon's rank sum test. a) Ion image of the feature m/z 428.2617, $1/K_0$=0.960 b) Ion image of the feature m/z 655.2736, $1/K_0$=1.272, which is reported as coproporphyrin III in the original publication.

3.2.3 Differential analysis with linear mixed effects models

Non-spatial fixed effects model For each feature, we fit the model

$$y_s = \mu_k + \varepsilon_s$$

where the response y_s is the intensity at pixel s, the fixed effect μ_k is the mean intensity in group k, $\varepsilon_s \sim \mathcal{N}(0, \sigma^2)$ is the random error at s.

Mixed effects model with spatial correlation For each feature, we also fit a mixed effects model with Matérn spatial correlation presented in Ospina *et al.* [2]. The authors found that the Matérn spatial model had a better fit than a non-spatial model on densely sampled spatial transcriptomics data. Since MSI data are similarly densely sampled as pixel grids, we also pursued this approach here. However, Matérn spatial model is not the only possible model and is not necessarily the best spatial correlation structure for all MSI data.

The Matérn spatial model represents pixel pair-wise correlation within each ROI as follows:

$$y_s = \mu_k + U_s + \varepsilon_s$$

where y_s is the intensity at s, μ_k is the mean intensity in group k, U_s is the Matérn random field at s, and $\varepsilon_s \sim \mathcal{N}(0, \sigma^2)$ is the random error at s. The Matérn random field was sampled from $\mathcal{N}(\mathbf{0}, \sigma_u^2 \mathbf{V})$, that the correlation matrix $\mathbf{V}$ is

$$V_{ij} = \mathrm{corr}(d_{ij}) = \frac{1}{2^{\nu-1}\Gamma(\nu)} \left(\frac{d}{\rho}\right)^{\nu} K_\nu \left(\frac{d}{\rho}\right)$$

where d is the Euclidean distance between pixel i and j, Γ is the Gamma function, K_ν is the modified Bessel function of the second kind with order ν, and ρ and ν are parameters. The parameter ν controls local smoothness. The special case $\nu = 0.5$ (used in this study) simplifies the correlation to exponential decay with respect to distance, which is common for stable estimation and easy optimization:

$$\mathrm{corr}(d_{ij}) = \exp\left(-\frac{d}{\rho}\right)$$

Parameters of the model, in particular the variance σ^2 and covariance $\sigma_u^2 \mathbf{V}$ are estimated using restricted maximum likelihood. The standard error of μ_k is calculated approximately based on the likelihood function. Finally, the null hypothesis $H_0 : \mu_k - \mu_{k'} = 0$ was tested using Wald test, which compares the ratio of the estimated difference in means to their standard error to the Standard Normal distribution.

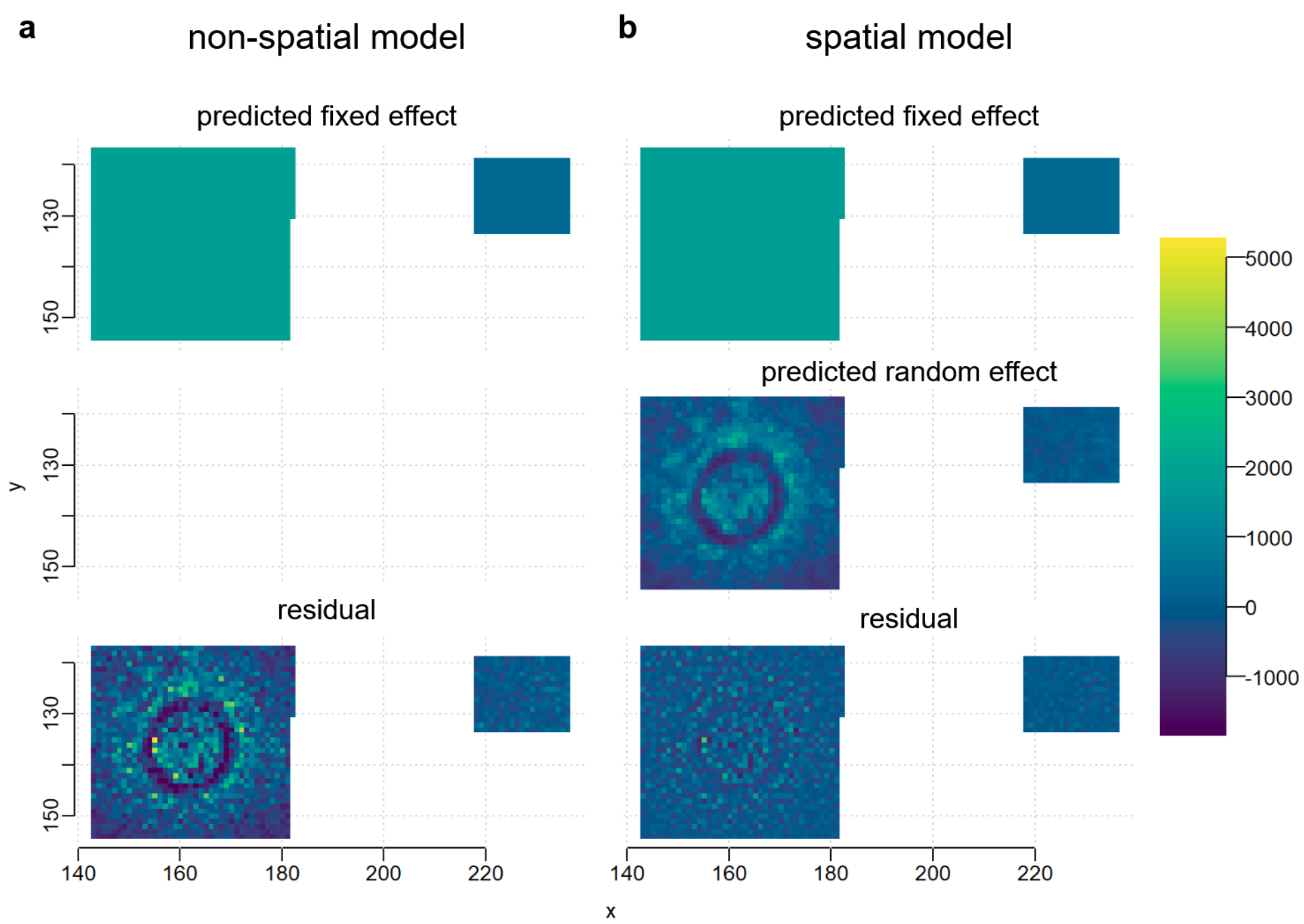

Figure S7: **Microbial natural products dataset:** comparison of mixed effects linear model fit for feature m/z 562.115 ($1/K_0$=1.16). (a) Predicted fixed effect and residual maps of the non-spatial model. (b) Predicted fixed effect, predicted random effect and residual maps of the spatial model. With the Matérn spatial random effect, the residuals became less spatially correlated and closer to Gaussian noise, suggesting a better fit.

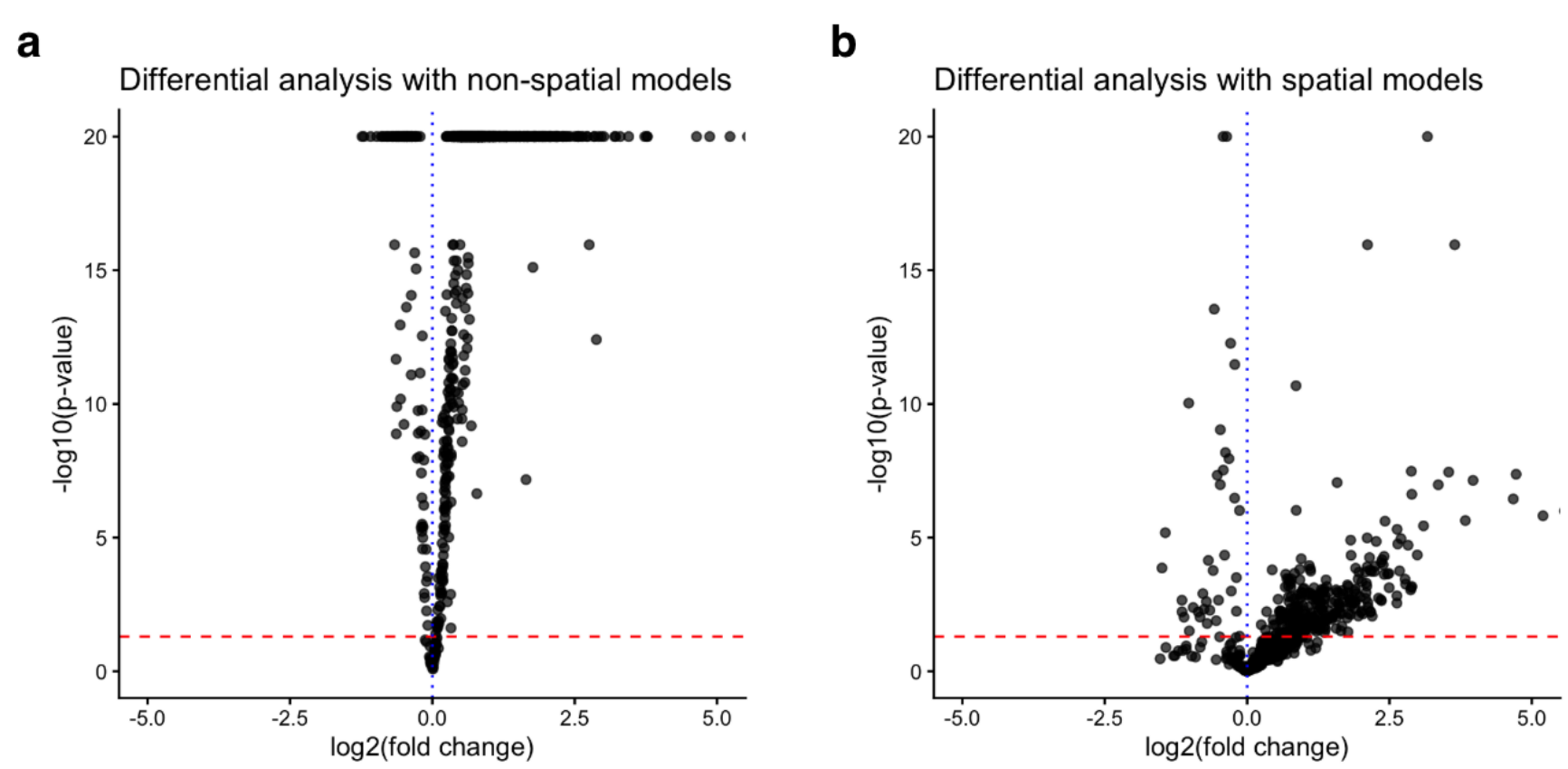

Figure S8: **Microbial natural products dataset:** volcano plot of differential abundance analysis with the (a) non-spatial model, a liner model with only group label as the fixed effect (b) spatial model, with group label as the fixed effect and Matérn spatial correlation as the random effect. Many p-values of the non-spatial models are extremely small, and for the same feature the p-values from the non-spatial model tend to be smaller than those from the spatial model, which is consistent with Ospina's observation. The potential reason is that the variance across pixels was underestimated in non-spatial modeling, resulting in artificially smaller p-values (type I error).

3.2.4 Comparison of differential analysis between processing with and without ion mobility

For technical validation of the benefits of ion mobility in terms of differential analysis, we also processed raw data while ignoring ion mobility dimension using TIMSCONVERT and Cardinal. The processing workflows with and without ion mobility are contrasted in **Figure S10**.

Processing while ignoring ion mobility In the TIMSCONVERT workflow, the data were first converted into imzML without any modification. The imzML was then processed in Cardinal, including binning signals from isobaric ions, ROI selection and peak processing. The resulting data allowed spatial analyses in Cardinal, such as image segmentation and principal components analysis. The fundamental limitation of this workflow is that it cannot take advantage of the ion mobility dimension. Moreover, the peak processing algorithms in Cardinal are designed for 1D mass spectra, and collapsing 2D m/z-ion mobility data by binning causes accuracy loss. Finally, the size of unprocessed imzML is large (25GB in Case study 2), consuming large anounts of storage space and processing time.

Ion mobility-aware processing Alternatively, TIMSImaging accessed the raw data and performed processing directly as detailed in Case study 3 Vignette. It then exported continuous, processed imzML with ion mobility, in which isobaric ions were written as separate features. The resulted imzML was much smaller (145MB and 378 peaks in Case study 2). It was ready for spatial analysis in Cardinal, and enabled other downstream analysis such as differential abundance analysis and CCS-based annotation.

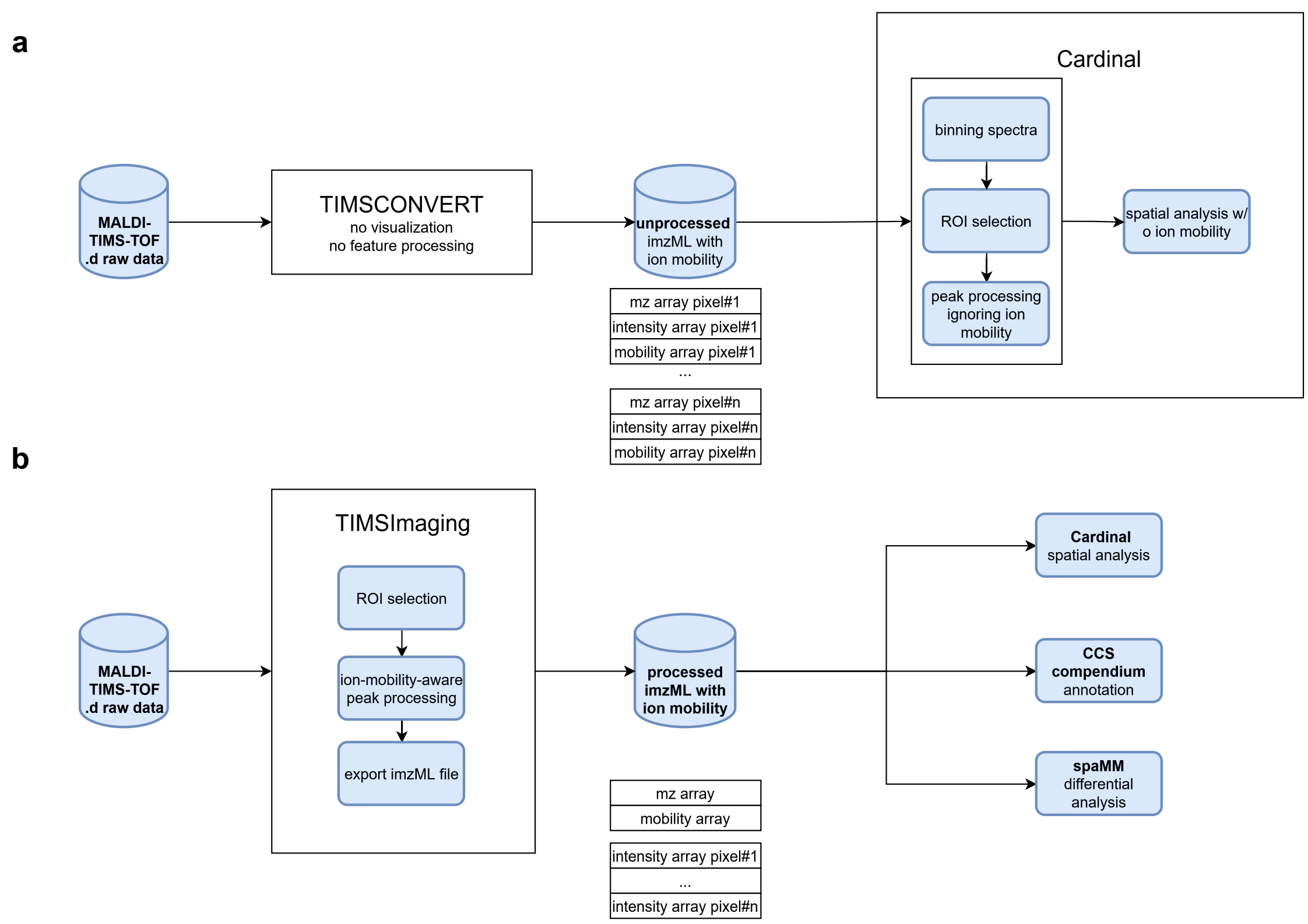

Figure S9: **Comparison of spectral processing strategies.** (a) Without ion mobility. (b) With ion mobility.

3.3 Case study 3: Lipids annotation of mouse skin tissue

3.3.1 Experimental details

Skin from mouse forepaws were snap-frozen in optimal cutting temperature medium and 5 μm cryosections were cut on super-frost microscopic glass slides covered with 2,5-dihydroxybenzoic acid (DHB) matrix. MALDI-TIMS-MS1 imaging dataset was acquired in positive mode using a timsTOF fleX mass spectrometer (Bruker Daltonics, Bremen, Germany) at a pixel size of 20×20 μm in the m/z range 300–1350 and 1/K0 range 0.70-1.96 V · s/cm2. The data contains 65052 pixels in total and is over 3 GB in raw (.d) format.

3.3.2 Data processing with TIMSImaging

The raw data were processed using parameters detailed in Case study 3 Vignette, resulting in 930 features. The CCS values were then calculated from m/z and $1/K_0$ using a linear model, the results differed no more than 0.3% from the values calculated by Bruker's internal function. The feature list was exported to CSV for CCS Compendium annotation, and the processed data was exported to imzML for METASPACE annotation.

3.3.3 Annotation using CCS Compendium database

The feature list was queried against a subset of CCS Compendium database (super class=lipids and lipid-like molecules, charge=+1) with 5 ppm m/z tolerance, then hits were ranked by CCS error.

3.3.4 Annotation exported imzML with METASPACE

The imzML output of TIMSImaging was uploaded to METASPACE and annotated using the following parameters:

**Name**	**Value**
Metabolite database	Lipid Maps 2017-12-12
Adducts	[M+H], [M+K], [M+Na]
Analysis version	v1 (Original MSM)
m/z tolerance	3 ppm

Table S2: **Mouse skin lipids dataset:** Parameters used for METASPACE annotation

The annotation project is available at `https://metaspace2020.org/dataset/2026-01-07_13h02m29s`. 96 features were annotated with FDR=10% and the feature list was downloaded for comparison with CCS-based annotation.

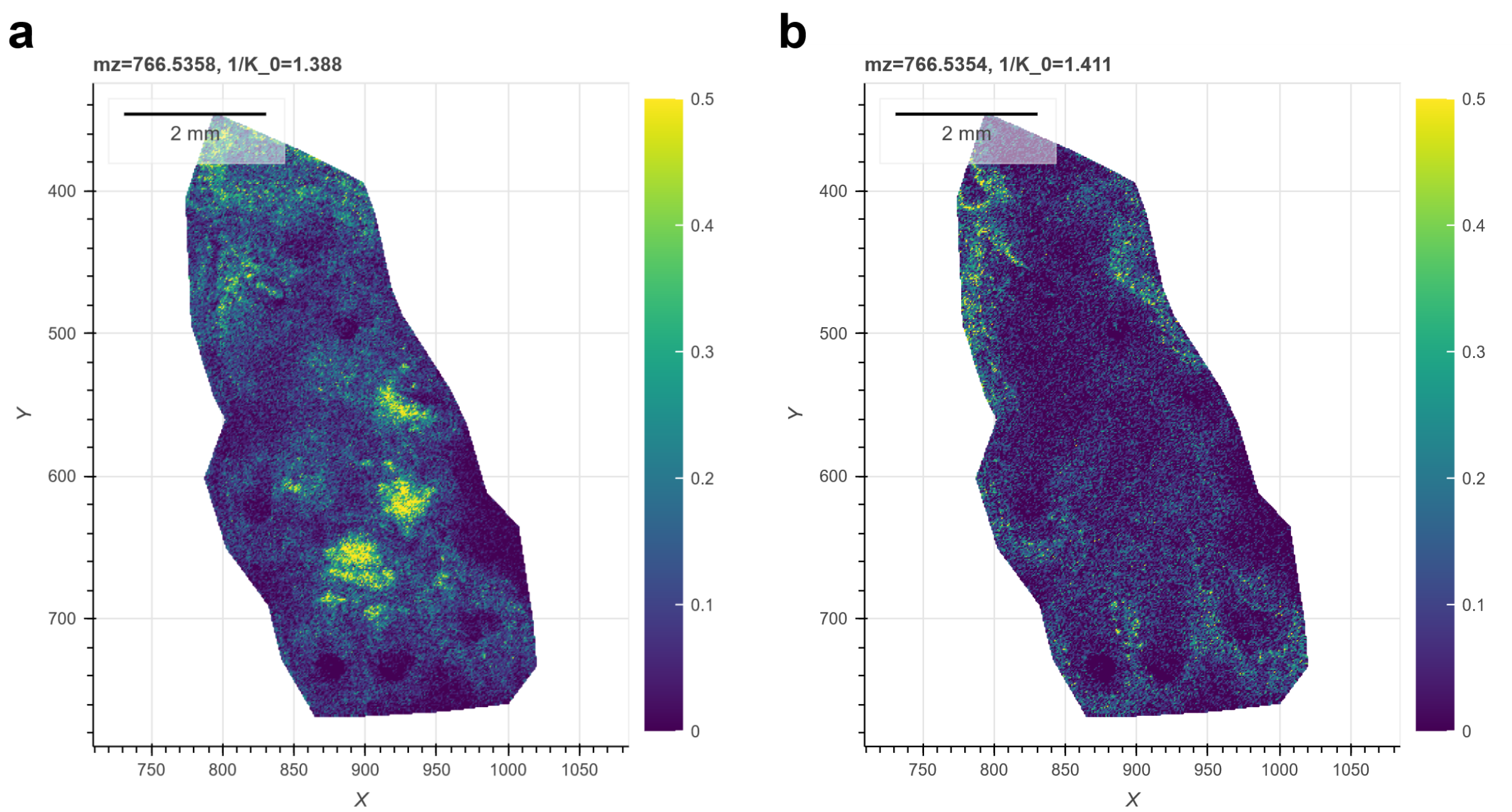

Figure S10: **Mouse skin lipids dataset:** ion images of isomeric features annotated as different lipids. (a) image of the feature annotated as PE(36:02) (b) image of the feature annotated as PE(18:1/18:1)(del9-cis). There is ambiguity in CCS Compendium itself, that PE(36:02) has multiple isomers with different double bond locations and configurations.

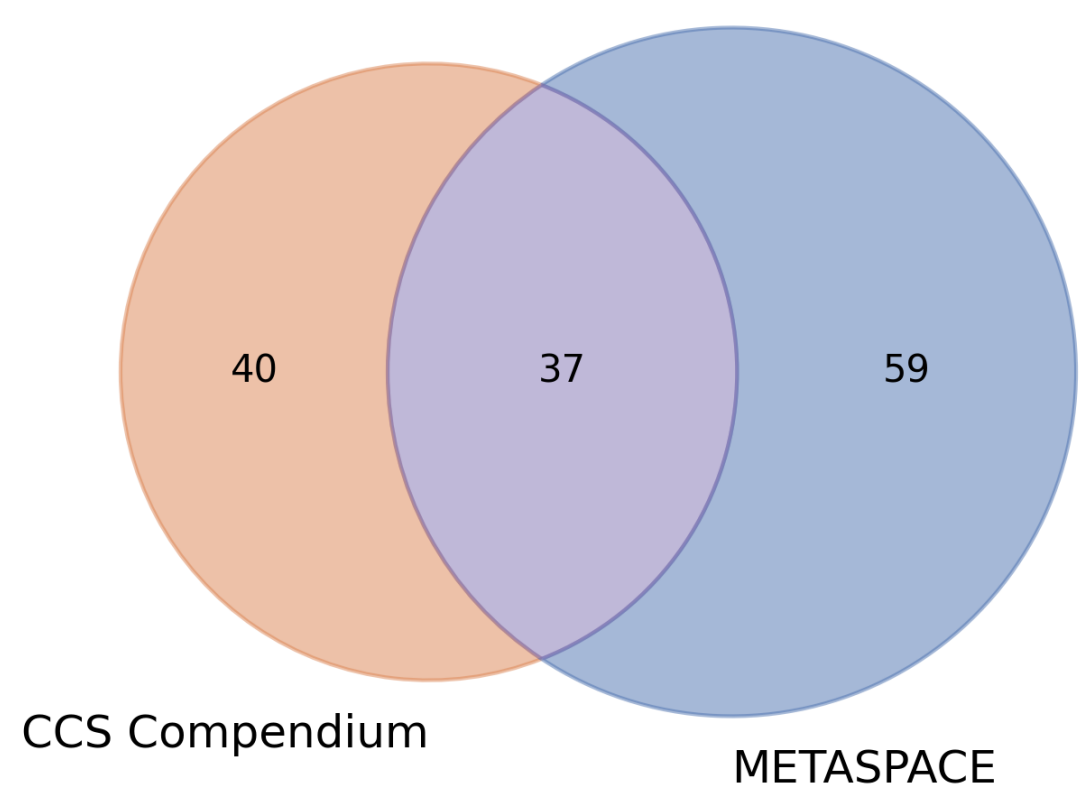

Figure S11: **Mouse skin lipids dataset:** Venn diagram of features annotated with CCS Compendium and METASPACE. The features only annotated by METASPACE are due to much larger size of database. The features only annotated by CCS Compendium are rare adducts not covered in METASPACE search, and possibly with low METASPACE scores.

Table S3: Mouse skin lipids dataset: top 20 CCS match annotations with their theoretical and measured m/z and CCS values, feature IDs, and associated mass and CCS errors.

**Compound**	**Formula**	**Adduct**	**Theor.** m/z	**CCS**	**Feature ID**	**Meas.** m/z	**Meas. CCS**	**Mass error (ppm)**	**CCS error (Å^2)**	**CCS dev. (%)**
1,2-Diarachidoyl-sn-glycero-3-phosphocholine	$C_{48}H_{96}NO_8P$	[M+Na]	868.6772	310.7	776	868.6761	311.0	-1.3	0.3	0.09
PC (18:1(9Z)/16:0)	$C_{42}H_{82}NO_8P$	[M+Na]	782.5676	292.1	621	782.5675	291.5	-0.2	-0.6	0.19
1,2-Distearoyl-sn-glycero-3-phosphoethanolamine	$C_{41}H_{82}NO_8P$	[M+Na]	770.5676	289.8	599	770.5672	289.2	-0.5	-0.6	0.19
1,2-Distearoyl-sn-glycero-3-phosphocholine	$C_{44}H_{88}NO_8P$	[M+Na]	812.6146	298.6	687	812.6110	299.2	-4.4	0.6	0.20
PE 36:01	$C_{41}H_{80}NO_8P$	[M+2Na-H]	790.5339	284.9	637	790.5372	285.5	4.2	0.6	0.21
1,2-Dipalmitoyl-sn-glycero-3-phosphocholine	$C_{40}H_{80}NO_8P$	[M+Na]	756.5520	286.2	572	756.5522	287.3	0.3	1.1	0.37
1-Palmitoyl-2-oleoyl-sn-glycero-3-phospho-ethanolamine	$C_{39}H_{76}NO_8P$	[M+Na]	740.5207	281.7	544	740.5220	280.1	1.8	-1.6	0.57
1,2-Dilauroyl-sn-glycero-3-phosphocholine	$C_{32}H_{64}NO_8P$	[M+H]	622.4448	260.6	357	622.4475	262.2	4.3	1.6	0.61
PE (18:1/18:1)(del9-cis)	$C_{41}H_{78}NO_8P$	[M+Na]	766.5363	285.8	591	766.5354	287.8	-1.2	2.0	0.69
PE 36:02	$C_{41}H_{78}NO_8P$	[M+Na]	766.5363	281.0	590	766.5358	283.1	-0.7	2.1	0.75
PC (18:1/18:1) (del9-trans)	$C_{44}H_{84}NO_8P$	[M+H]	786.6013	293.3	628	786.5983	295.6	-3.8	2.3	0.78
1,2-Diheptadecanoyl-sn-glycero-3-phospho-ethanolamine	$C_{39}H_{78}NO_8P$	[M+Na]	742.5363	282.1	547	742.5372	279.6	1.3	-2.5	0.87
PE (18:1/18:1)(del9-cis)	$C_{41}H_{78}NO_8P$	[M+Na]	766.5363	285.8	590	766.5358	283.1	-0.7	-2.7	0.94
PE 34:01	$C_{39}H_{76}NO_8P$	[M+Na]	740.5207	277.1	544	740.5220	280.1	1.8	3.0	1.08
Cer 42:02	$C_{42}H_{81}NO_3$	[M+H-H_2O]	630.6189	275.4	370	630.6214	278.6	4.0	3.2	1.14
PE 39:02	$C_{44}H_{84}NO_8P$	[M+2Na-H]	830.5652	293.0	723	830.5631	296.9	-2.5	3.9	1.34
PE 36:01	$C_{41}H_{80}NO_8P$	[M+Na]	768.5520	283.3	595	768.5523	287.2	0.4	3.9	1.38
PC (18:1(9Z)/16:0)	$C_{42}H_{82}NO_8P$	[M+H]	760.5856	287.9	578	760.5828	291.9	-3.7	4.0	1.40
PE 36:03	$C_{41}H_{76}NO_8P$	[M+Na]	764.5207	278.7	585	764.5214	282.7	0.9	4.0	1.44
PC (16:1/16:1) (del9-cis)	$C_{40}H_{76}NO_8P$	[M+H]	730.5387	278.4	527	730.5376	282.6	-1.5	4.2	1.52

References

[1] A. C. George, I. Schmitz, F. Rouviere, S. Alves, B. Colsch, S. Heinisch, C. Afonso, F. Fenaille, and C. Loutelier-Bourhis. Interplatform comparison between three ion mobility techniques for human plasma lipid collision cross sections. *Anal Chim Acta*, 1304:342535, 2024.

[2] O. E. Ospina, A. C. Soupir, R. Manjarres-Betancur, G. Gonzalez-Calderon, X. Yu, and B. L. Fridley. Differential gene expression analysis of spatial transcriptomic experiments using spatial mixed models. *Sci Rep*, 14(1):10967, 2024.
